# The Medical Research Council's Annual Report

**Published:** 1921-01-08

**Authors:** 


### A YEAR'S PROGRESS.

### The Medical Research Council's Annual Report.

The report covering the year 1919-20 includes an enormous list of investigations which are being carried out on behalf of the Council, and it is impossible here to mention more than one or two outstanding points in the series. The report itself does not, in fact, enter into any great detail as to results and prospects, except where a considerable degree of finality has already been attained. Its chief value to the practical health worker lies, therefore, more as a book of reference on the men and institutions particularly devoting their energies to the various types of investigation than as an instructive scientific volume. But, nevertheless, it shows, reading between the lines, a wonderful record of steady progress; and it is needless to urge how vitally this research work affects at every point the great issues of health and happiness in the race.

### The National Institute.

Progress with the National Institute for Medical Research is noteworthy. Occupation of the building and grounds at Mount Vernon, Hampstead, was recovered in June 1919. This building had been purchased in 1914 and lent, first to the War Office, and later to the Air Ministry, for hospital purposes during the War. As quickly as possible the work of equipping the building as a research institute was put in hand, but contrary to the general hopes on the matter, labour difficulties and a variety of other causes combined to delay the alterations, and by the end of 1919 it had been found possible to install only the Departments of Statistics and of Publications in their new home. Meanwhile, those of Biochemistry and Pharmacology and of Applied Physiology continued their tenancy at the Lister Institute, and the Department of Bacteriology and Experimental Pathology remained at St. Mary's Hospital. In April 1920 it was, however, decided to concentrate the work of all the above in the Hampstead buildings. Although equipment is still somewhat incomplete, the few months' experience already gained shows, apparently, all the expected advantages of a close association of workers in the different technical branches of research. The Council may justifiably look with confidence for excellent results from this free association, but it is a point to be remembered, however, that it is not the intention of the Council to draw any sharp line of division between scientific workers within the Institute, and those working in various research schemes at universities, hospitals, etc.

### Individual Sections.

As might be expected, the work involved in the removal and reopening of these departments has taken up a considerable portion of the staff's time, and it is not surprising that the year does not show many long and completed researches. There are, however, one or two particularly interesting subjects under investigation. In conjunction with Dr. Leonard Hill, there is a series of experiments on the influence of chilled surfaces upon bacteria suspended in a fine spray, the everyday applications of which will be obvious to most who have experienced the recent infective outbreaks. Two well-known disease-producing germs are also being dealt with by the direct method, and large quantities of *B. typhosus and B. tuberculosis* are being prepared with the object of isolating and exactly analysing their constituent proteins, opening thus a possibility of finding out other essential properties of disease germs, besides their "cultural reactions," which are certainly neither as definite nor as specific as scientists once thought them to be.

In the Biochemical department further attention is being devoted to the active principles of the secretion produced by the ductless glands of the animal body. The importance of this work will be apparent to those who take interest in the variety of therapeutic fads by which the medical profession is at times overwhelmed. Note was made a short time ago in these pages on the vogue of "organo-therapy," and though no one will deny an immensity of good *may*, in certain deficiency cases, be done by administration of ductless gland extract, yet there is a positive mountain of ignorance still to be overcome before the exact principles of these vital issues are completely understood.

Further on, an interesting note is provided in connection with the wholesale treatment of malarial infections by quinine. From numerous sources evidence has accumulated during the war that quinine, while controlling the immediate symptoms of both forms of "Tertian" malaria and effecting a radical cure in a very high proportion of cases of infection with the "Malignant Tertian" parasite, produces this *complete cure* in relatively few cases of the so-called "Benign Tertian" fever. As a result there are now in this country, and on the roll of the Ministry of Pensions, large numbers of men who are still infected with "Benign Tertian" malaria and liable to periodic relapses. Work by Major Acton strongly suggests, however, that other alkaloids of cinchona, and in particular quinidine and cinchonidine, will prove to be much more effective for the radical cure of this condition than quinine.

Another investigation has gone into the actual nature and correct treatment of the circulatory and cardiac failure which frequently occurs early in diphtheria and is, on occasions, so severe that administration of anti-toxin alone is without avail. Unfortunately, we are not yet provided with details of the method, but it undoubtedly suggests the saving of many lives.

The composition and standardisation of salvarsan derivatives for the treatment of syphilis are still

**A Year's Progress**—(*continued*).

receiving abundant attention; and upon the same organised principles a large number of drugs are being dealt with, the exact strength of which can be ascertained only by biological experiment and not by any simple chemical estimation. This part of the work is singularly necessary since, although a few of the first-class manufacturers have themselves carried out this work in the past (and done it extremely well), yet it is a matter which calls for centralised control.

In the Physiological section it may be noted that the true principles and factors in open-air treatment and "heliotherapy" are to be carefully investigated upon lines already taken up by Dr. Leonard Hill, who has, incidentally, devised yet another thermometric instrument. His Katathermometer has already achieved no little fame and distinction. In the Statistical department we note that a systematic study of the incidence of rheumatic fever is being undertaken and it is a useful step. This baffling, and unfortunately most crippling, condition, has too long run rife among our younger population and anything leading to a true elucidation of its causal factors should be invaluable.

The Clinical Teaching Units.

So might one run many times through the report, picking out each time some new point here, some fascinating possibility there, but in this note, having referred simply to the Council's own departments, let us in conclusion consider the experimental medicine and the research work of the new clinical units in the hospitals.

The policy of the Council was sketched in their last annual report. They gave reasons for not instituting, for the present at least, special beds for research purposes at their own Institute or elsewhere and for gaining, if possible, most of the advantages of special research wards by suitable arrangements made at existing hospitals. There research done can be brought into the correct relation to medical education and the higher teaching of medicine. Also in the last report stress was laid upon the facts that, "the traditions and practice of the past have not produced any school of younger men trained in advance by research in the science of experimental medicine. At present there is a dearth of such men, which has been lately revealed in the most striking way at London and at other university centres." With this, therefore, in view, the Council have given whatever co-operation lay in their power to the prospective formation of what, for want of a better name, are being called the hospital "units." Such arrangements can now be seen at University College, St. Bartholomew's, The London, St. Thomas's, and elsewhere. It is a new phase, but, so far, a sound one. The Council and the units are deserving of full national encouragement.

